# Protruding Objects in the Membrane Lung Outlet May Increase Thrombogenicity: Fluid Dynamical Insights

**DOI:** 10.1097/MAT.0000000000002533

**Published:** 2025-08-26

**Authors:** Frida Nilsson, Monica Emendi, Lars Mikael Broman, Lisa Prahl Wittberg

**Affiliations:** From the * FLOW, Department of Engineering Mechanics, Royal Institute of Technology (KTH), Stockholm, Sweden; † ECMO Centre Karolinska, Astrid Lindgren’s Children’s Hospital, Karolinska University Hospital, Stockholm, Sweden; ‡ Department of Physiology and Pharmacology, Karolinska Institute, Stockholm, Sweden.

**Keywords:** ECMO, oxygenator, membrane lung, thrombogenicity, large eddy simulations, computational fluid dynamics, scanning electron microscopy

## Abstract

Thrombosis in extracorporeal membrane oxygenation (ECMO) circuit components remains a challenge. Besides blood state and surface properties, flow plays a critical role in hemostasis. In this work, we aimed to study the fluid dynamics of a membrane lung (ML) outlet due to its complex design with pins protruding into the blood flow stream (temperature sensor and cap of purge line), with respect to the potential risk of flow-induced coagulation activation. Large eddy simulations were carried out for blood flow of 1 and 4 L/min. Recirculation bubbles and strong vortical structures developed in this geometry. These flow structures were similar to characteristics of flow past bluff bodies, which facilitate entrapment of platelets that may be activated by the elongational shear rates (> 2,000 s^−1^), observed near the surface of the temperature sensor for the 4 L/min case. Moreover, a thrombus, extracted from an ECMO circuit, was analyzed by scanning electron microscopy. It is concluded that a review of devices used for ECMO with auxiliary objects protruding into the bloodstream is warranted for improvement of design to reduce the risk of blood trauma and coagulation activation.

The membrane lung (ML or oxygenator) is one of the circuit components used in extracorporeal membrane oxygenation (ECMO) associated with blood trauma and consequent onset of the coagulation cascade. The incidence of thrombus formation, reportedly 19% to 36%,^1^ is influenced by factors such as ML type, coating material, local anticoagulation protocol, patient’s primary disease, pump design, ECMO blood flow, *etc*.^2–6^ Development of clots in the ML or circuitry may induce hemolysis with secondary effects of increased risk of organ failures due to oxidative stress, and further increased coagulation, and inflammation.^7^ This may switch into a bleeding problem if coagulation proteins and platelets are consumed.

It is well known that low shear stress but prolonged residence times (stagnant flow), high shear stress (separated flow and shear layers), and temporal and spatial gradients (turbulent flow) are prone to increase the risk of thrombus formation.^8–10^ In the components of the ECMO circuit, all these flow characteristics develop. Regions of stagnant flow are commonly found in geometries including sudden steps, as in the inlet and outlet of the connector, as well as the Luer-lock connectors. Several studies,^11,12^ as well as clinical experience, have pointed out the risk of fibrin deposition and thrombus formation in connectors, a feature that can be mitigated by redesigning the geometry as recently proposed by Bresette *et al*.^13^ Similar geometries are commonly found in the adaptors between ECMO devices and tubing, for example, pump, ML and cannulae.

This work focuses on the Quadrox ID Adult ML (Getinge, Rastatt, Germany) due to the complex configuration of the auxiliary objects placed in the outlet of the ML. The objective was to characterize the resulting fluid dynamics and assess the potential risk of flow-induced coagulation activation.

## Methods

### Geometry Reconstruction

**Figure 1.**
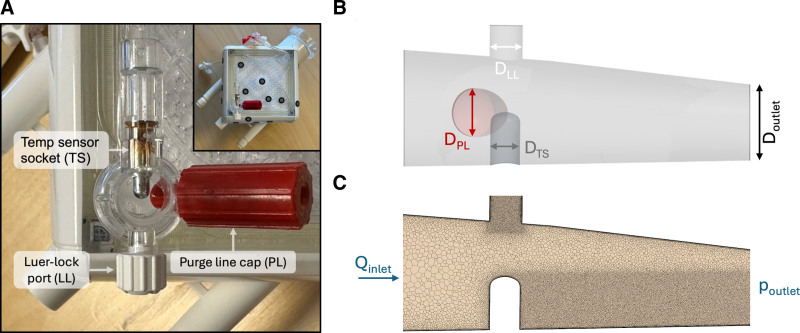
Geometry and details of the computational domain. **A**: Quadrox iD Adult membrane lung (Getinge, Rastatt, Germany) with detail of its outlet with temperature sensor socket (TS), Luer-lock port (LL), and the purge line cap (PL) (top view). **B**: Side view of CAD geometry used for simulations, with diameters (D_LL_ = 4 mm, D_PL_ = 5.9 mm, D_TS_ = 3.6 mm, D_inlet_ = 14.1 mm, D_outlet_ = 9.2 mm). **C**: Mesh detail with refinement of LL and of the wake behind TS. Boundary conditions (flow rate at inlet, Q_inlet_, and pressure at outlet, p_outlet_) are indicated.

The outlet port geometry of the Quadrox iD Adult ML was reproduced from caliper measurements and 3D surface scanning. The main geometrical features, as displayed in Figure 1, were:

- a temperature sensor socket (TS), which protrudes into the outlet port for half of the outlet port’s diameter (base diameter D_TS_ = 3.6 mm),
- a male cap for the purge line (PL), that is, a connector for priming or high-flow fluid replacement, with an inclination of 64° from the main outlet axis (base diameter D_PL_ = 5.9 mm),
- a Luer-lock port (LL) used to access the circuit for pressure measurement, blood sampling, *etc*.

### Numerical Methodology and Case-Set Up

Computational fluid dynamics (CFD) simulations were carried out using the commercial software Star-CCM+ (Version 2410). To resolve all flow scales, explicit large eddy simulations (LES) with a WALE subgrid scale (SGS) model were applied using a second-order bounded central scheme for the convective terms. To initialize the LES, SST *k*-*ω* Reynolds averaged Navier Stokes (RANS) was used. The blood was assumed to have a constant viscosity of 3.5 mPa • s and a density of 1,050 kg/m^3^. No slip boundary conditions were applied at all solid walls, including the perturbed items. At the inlet, a constant mass flow rate was applied, investigating two different ECMO flow rates of 1 and 4 L/min. The downstream pressure was set to zero at the outlet.

A polyhedral mesh was adopted with local mesh refinement in the wake regions behind the bluff bodies and inside the LL (Figure 1C). Bluff bodies refer to objects in the flow that cause the flow to separate abruptly, which can lead to the creation of vortices and turbulence behind the object. To allow the flow to develop and to reduce any boundary reflections, extrusions were added to both inlet and outlet. For the mesh sensitivity study, three different meshes were assessed: 3.5, 8, and 18.6 million cells (Figure S1, Supplemental Digital Content, https://links.lww.com/ASAIO/B623). To resolve the relevant flow structures, the medium mesh was used for the simulations.

### Flow Behind Bluff Bodies

From a fluid dynamics point of view, the TS socket and the PL cap act as bluff bodies in a confined pipe flow.^14,15^ The Reynolds number based on the cylinder diameter^16^ was calculated for TS and PL according to:


$$\begin{array}{l} Re_{TS}=\frac{\rho uD_{TS}}{\mu } \end{array}$$ (1)

$$\begin{array}{l} Re_{PL}=\frac{\rho uD_{PL}}{\mu } \end{array}$$ (2)

where ρ is the density, *u* is the velocity, *D*_TS_ and *D*_PL_ are the diameters of the TS and PL cap, respectively, and μ is the dynamic viscosity.

To characterize the vortex shedding, a fast Fourier Transform (FFT) of the local velocity was calculated for different probe points placed behind the bluff bodies, and the related Strouhal number^17^ was calculated according to:


$$\begin{array}{l} St_{TS}=\frac{fD_{TS}}{u} \end{array}$$ (3)

where *f* indicates the frequency of the shedding, obtained from the FFT.

### Analysis of the Fluid Strain Rates

The local rate of strain and rotation for a fluid element can be described by the symmetric and antisymmetric parts of the velocity gradient:


$$\begin{array}{l} \nabla u=\frac{1}{2}\left(\nabla u+\nabla u^{T}\right)+\frac{1}{2}\left(\nabla u-\nabla u^{T}\right)=D+W \end{array}$$ (4)

where **D** is the rate of strain and **W** is the rotation tensor. In accordance with Yeo *et al*.,^18^ the magnitude of the strain rate, $\dot{\gamma }$, and rotation rate, $\dot{\omega }$, is quantified by:


$$\begin{array}{l} \dot{\gamma }=\sqrt{2D:D},\;\;\dot{\omega }=\sqrt{2W:W} \end{array}$$ (5)

where “:” indicates the double dot product.

In regions with $\dot{\gamma }\gg \dot{\omega }$ the flow can be defined as elongational. Average values of $\dot{\gamma }$ and $\dot{\omega }$ were calculated for the last second of the simulation, and regions of elongational flows were defined where $\dot{\gamma }-\dot{\omega }>1000$.

### Comparison With the Quemada Viscosity Model

For the 1 L/min case, an additional LES was carried out considering a non-Newtonian Quemada model^19^ for blood viscosity, and the obtained results were compared to the ones obtained assuming a constant viscosity for blood. This was done to assess the sensitivity of the study to the modeling choices, and the 1 L/min case was chosen since lower shear rates were expected and thus more significant non-Newtonian effects.

### Clinical Material and Ethical Aspects

A thrombus was collected from a used ECMO circuit of a 57 year old woman after circuit removal. Sample collection was done in accordance with relevant regulations and guidelines, including informed written consent from the patient. (Approved by the Swedish Ethical Review Authority, Dnr 2019-06446 0409.)

### Sample Preparation for Scanning Electron Microscopy

Upon removal from the patient, the ECMO circuit was rinsed with tap water, and the thrombus was collected and put in a petri dish with saline solution (NaCl 154 mmol/L). The thrombus was then cut into 1 mm strands before fixation in 2.5% glutaraldehyde for 24 h. After fixation, samples were transferred into a 0.1 M phosphate buffer solution and refrigerated pending analysis. To prepare for scanning electron microscopy, the sample was critically point dried (CPD) using a Leica CPD300 (Leica microsystems, Wetzlar, Germany), and sputter-coated with 15 nm platinum (Quorum Technologies Laughton, UK). All images were collected with a field emission scanning electron microscope (FESEM) Merlin, manufactured by Zeiss.

## Results

### Fluid Dynamical Features

The Reynolds numbers for the bluff bodies Re_TS_ and Re_PL_, defined in Equations 1 and 2, were respectively equal to 231 and 349 for 1 L/min, 845 and 1,168 for 4 L/min. For flow around cylinders, this indicates a subcritical flow regime with transition to turbulence for 1 L/min and a turbulent vortex street for 4 L/min.^17^ The Reynolds number for the outlet pipe was equal to 1,087 for 1 L/min and 3,670 for 4 L/min.

The vortex shedding frequency of the TS for 1 L/min was equal to 11 Hz and the corresponding Strouhal number (Equation 3) was 0.19, whereas for 4 L/min, the former was equal to 42 Hz and 0.2, respectively.

**Figure 2.**
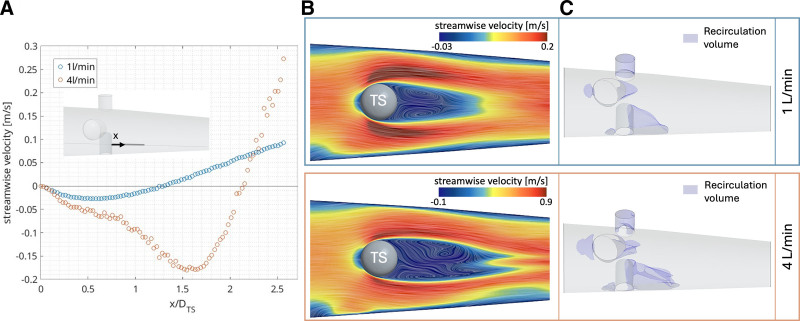
Wake length and recirculation volume. **A:** Streamwise velocity (averaged over the last second of simulation) calculated on the depicted line probe for 1 and 4 L/min. **B:** Average streamwise velocity on the temperature sensor socket (TS) mid-height plane, shown in **A**, with line integral convolution of the velocity field. **C**: Recirculation volume, defined as cells where the streamwise mean velocity is negative.

Recirculation bubbles formed in the wake behind the TS. The length of the wake (region behind bluff bodies with a negative mean streamwise velocity) at TS mid-height was equal to 1.3D_TS_ (4.7 mm) and 2.1D_TS_ (7.6 mm) for 1 and 4 L/min, respectively (Figure 2A), whereas the maximum length, located at the wall, was 7 and 9.5 mm, respectively. Recirculation zones also developed behind the PL and in the LL, although smaller as compared to the TS wake. Moreover, as expected in bluff body flows, the regions in front of the PL and TS showed stagnation points surrounded by slowly moving flow. These flow characteristics are depicted in Figure 2, B and C, and Figure S2, Supplemental Digital Content, https://links.lww.com/ASAIO/B623. The total recirculation volume was 2.4% and 3% of the ML outlet volume, for 1 and 4 L/min, respectively.

**Figure 3.**
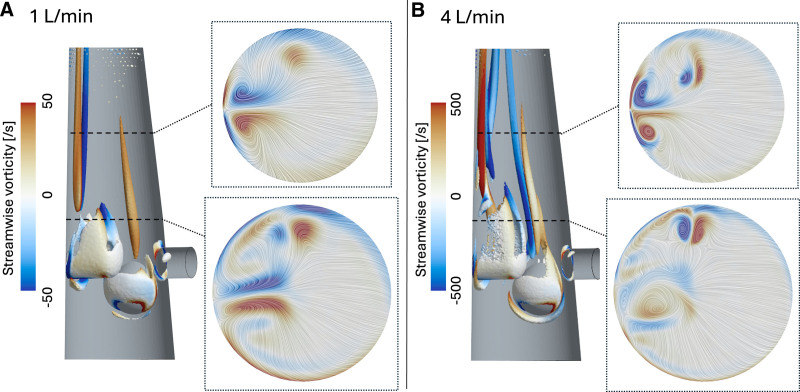
λ_2_ isosurface colored by average streamwise vorticity, with details of two transverse planes where line integral convolution of velocity is shown along with values of vorticity, for 1 L/min (**A**) and 4 L/min (**B**). Two different values of λ_2_ and two different scales of vorticity were used for (**A**) and (**B**) for visualization purposes.

The main vortical structures that characterize the flow in the ML outlet were a pair of counter-rotating vortices forming behind the bluff bodies from their base and extending until the outlet of the domain, and horseshoe vortices^20^ that developed in front of the bluff bodies, near their base. These structures, shown in Figure 3 A and B, were stronger and presented higher values of vorticity for 4 L/min compared to 1 L/min.

**Figure 4.**
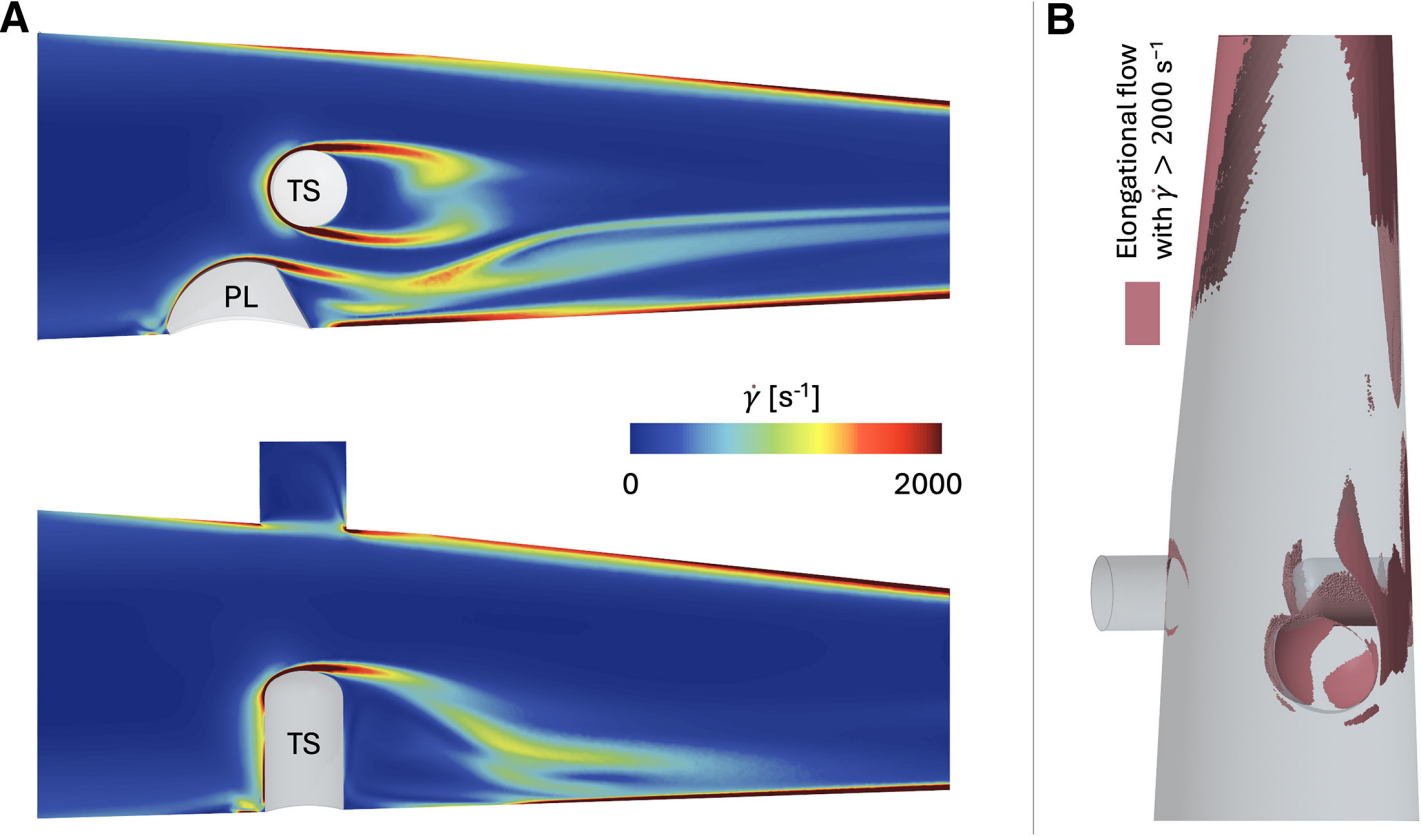
Fluid strain rates. **A**: Average values of $\dot{\gamma }$ on two perpendicular planes for the 4 L/min case. **B:** Volume of elongational flow with $\dot{\gamma }$ above 2,000 s^−1^ for the 4 L/min case.

Figure 4 shows, for the 4 L/min case, values of $\dot{\gamma }$ in two perpendicular planes and regions of elongational flow with $\dot{\gamma }$ above 2,000 s^−1^. The volume of elongational flow was equal to 3.9% of the total volume of the CFD domain, whereas the volume of elongational flow with $\dot{\gamma }$ above 2,000 s^−1^ was 2.3%. The values of $\dot{\gamma }$ on the upstream surface of the TS were above 10,000 s^−1^.

For 1 L/min, the highest values of $\dot{\gamma }$ were in the same location, with a maximum value of 2,800 s^−1^ (Figure S3, Supplemental Digital Content, https://links.lww.com/ASAIO/B623).

### Scanning Electron Microscopy Analysis of the Thrombus

**Figure 5.**
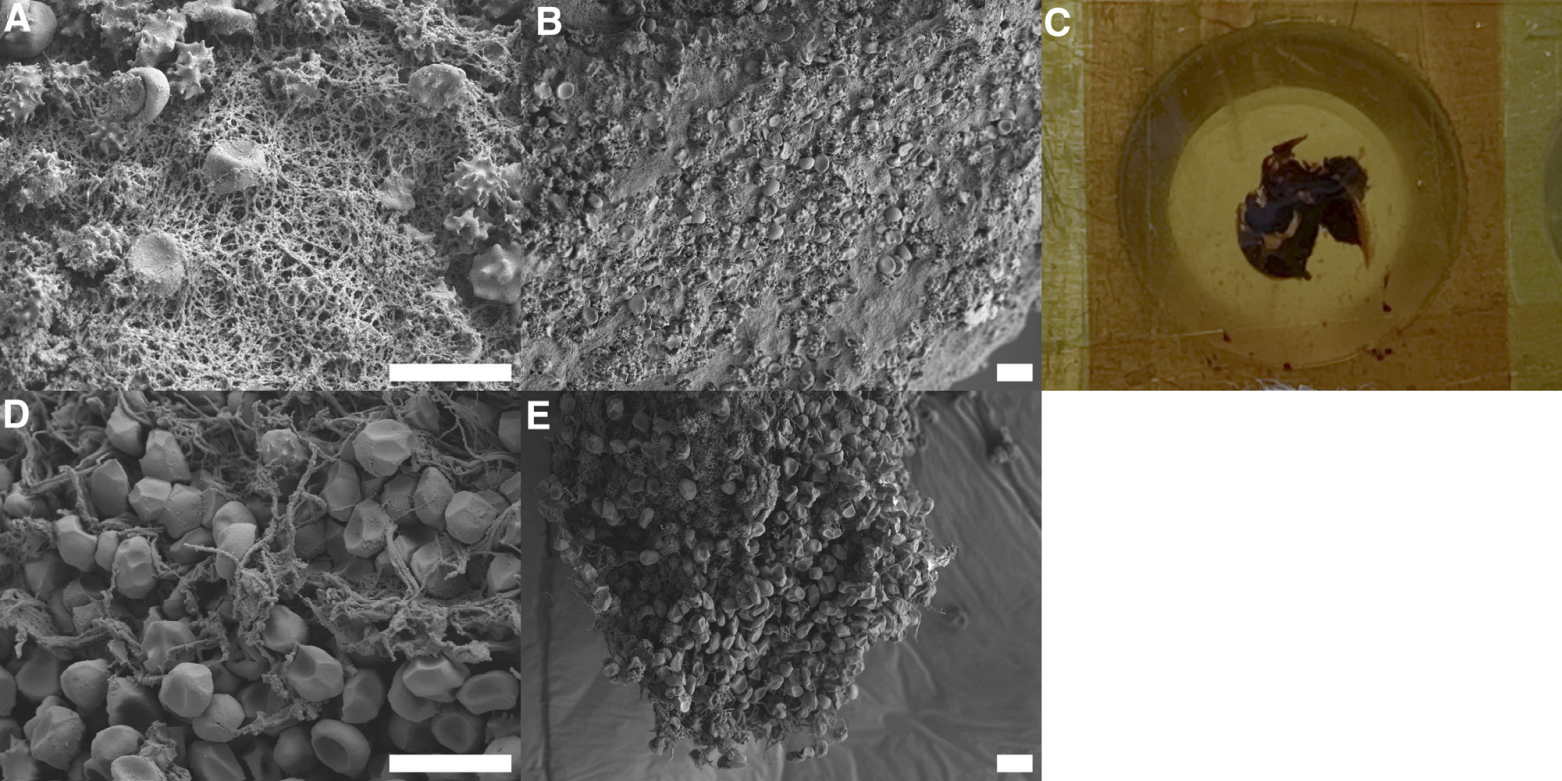
Representative scanning electron microscope images and a picture of the thrombus. **A, B**: Regular red blood cells and echinocytes caught in a fibrin mesh covered in platelet fragments. **C**: Piece of thrombus in a sample rig. **D, E**: Interior structure on a fractured surface with polyhedrocytes and regular red blood cells (RBCs) in a loose fibrin mesh. The scale bars correspond to 10 μm.

High-resolution scanning electron microscopy (SEM) was used to examine the interior and exterior structure of the thrombus found at the outlet of the ML, studying both the general and cell morphology. Around 50 images were captured in different regions of the thrombus. Imaging demonstrated that the thrombus was predominantly composed of fibrin and red blood cells covered in cell fragments, especially at the thrombus surface. The red blood cells were of different morphology, showing regular, echinocytes, and polyhedrocytes (Figure 5). The red blood cells were encapsulated by the fibrin mesh and had been pushed into tight bundles, morphing them into polyhedrocytes at the core of the thrombus.

## Discussion

This CFD analysis of an ML outlet showed regions of recirculation and stable vortical structures developing behind the TS and PL, in agreement with the fluid dynamics features that characterize the flow past bluff bodies.^15–17^ These flow features can facilitate the entrapment of platelets, as highlighted by previous works. Yang *et al*.^9^ showed the *in vitro* formation and growth of a thrombus in the recirculation region of a backward-facing step geometry: the larger the recirculation region, the larger the formed thrombus. Moreover, according to Bluestein *et al.*^21^ the shed vortices forming behind the leaflets of a mechanical heart valve promoted the formation of larger platelet aggregates, thus increasing the risk of free emboli formation. In addition, the LL, for which recirculation zones forms, is also a potential risk of thrombus formation as shown by previous studies.^12,22,23^

The flow directly dictates the forces acting on the fluid components. In this work, the fluid strain rate analysis revealed values above 10,000 s^−1^ located near the TS surface as well as areas of elongational flow with shear rate values above 2,000 s^−1^. According to Yeo *et al.*,^18^ von Willebrand factor (vWf), a mechanosensitive blood glycoprotein, believed to mediate thrombosis in high shear conditions, may unfold at elongational shear rates as low as 2,000 s^−1^ and thus enable binding to and activating platelets. Further, the combination of the PL and TS constitutes a major occlusion (~33% for the TS) of the cross section, posing issues like those of a stenosis in a vessel.^24^

The sensor pocket material for this device (Quadrox iD) was coated by a resin. However, uncoated stainless-steel surfaces are known to activate coagulation as recently reported. In an experimental work by Han *et al.*^25^ obstructive pins of different materials in a tube perfused at high shear rate (>5,000 s^−1^) showed stainless steel to be the most thrombogenic.

In this context, it should be noted that several of the MLs used today for ECMO were originally designed for extracorporeal circulation (ECC, heart-lung machine; approval 6 h). After change of membrane polymer and adding coating material, the basic designs (with TS, purge line and additional LL ports) could be approved for long-term use, enabling extension to ECMO. This allowed for marketing the same design to both ECC and ECMO. Newly approved redesigned MLs for ECMO, cleared of any unnecessary connections/features, are available (CARL, HERO; Resuscitec, Freiburg, Germany), with only one LL connector on the top of the ML and one on the arterial side before the blood is funneled towards the outlet. Further, MLs primarily designed for ECMO without TS or PL attributes are available for adults (Quadrox PLS; Getinge; Cardiohelp; Getinge; Nautilus; Medtronic, Tolochenaz, Switzerland; all with one LL on the venous side, on the arterial ML tank and on the outlet). For other designs, the PL does not protrude into the bloodstream. Instead, the PL causes a cavity, resulting in a similar flow behavior as induced by the LL. More adult MLs are available, but these have not been physically available for the authors, nor accessible via open source or other published material. However, several of these seem to have a TS in the bloodstream and typically a PL with a non-protruding cap as described above.

Depending on the needs of the patient, the used volume flow may vary. In this work, two different working conditions were studied: 1 L/min (for adults during trial-offs in the waning phase from ECMO) and 4 L/min (average flow rate during basic adult ECMO support). The recirculation volume was larger, the stresses were higher, and thus the thrombotic risk was greater in the 4L/min case, which is also closer to the working conditions often used in clinics. Another effect that influences the flow structure development is blood viscosity. A comparison between applying a non-Newtonian Quemada model and a Newtonian viscosity model showed that the viscous effects were underestimated using a constant viscosity. This indicates that the results shown here are less provoking than what could be expected from blood, especially in regions such as the wake forming behind the TS, also including strong shear layers between the slowly moving core behind the bluff body and the free stream, where a non-Newtonian model would imply higher viscosity and thus enhancing the shear layer as well as the recirculation zone (Figure S4, Supplemental Digital Content, https://links.lww.com/ASAIO/B623).

The experimental study of a thrombus collected in the outlet of the ML showed a dense fibrin shell around tightly packed polyhedrocytes. It has been shown that polyhedrocytes are formed from mechanical compression by activated platelets pulling on the fibrin mesh.^26,27^ In the current work, the fibrin mesh itself was covered with cell fragments that may stem from disintegrated platelets that were part of the clot contraction at an earlier stage of the thrombus growth process. Tutwiler *et al.*^28^ also showed that clot contraction benefits from high platelet concentrations in combination with a low hematocrit. Compared to normal values, the patient in this study had a hematocrit of ~30%, and a platelet count that rose from “low” to “normal” throughout the course of the treatment (120–180 × 10^9^/L). However, for a typical ECMO patient, both these values are considered as high at our ECMO Centre. The results indicate that platelets were highly involved at the onset of the clotting process. Moreover, the impact or presence of the phenomenon clot contraction on artificial surfaces is yet unknown.

The current study aimed at pinpointing the consequences of design choices on fluid dynamics and therefore potential risk of flow-induced coagulation activation, focusing on one ML’s outlet. The geometrical features of the studied ML are also present in other MLs on the market: PL (cavity instead protruding cap), TS, and LL (HiLite 7000LT; Xenios AG, Heilbronn, Germany); or TS and PL/LL (with protruding cap) (CM08 PMP; Chalice, Worksop, UK). For certain designs, the LL is angled; however, recirculation zones with stagnant flow are still expected to be generated. A cap design not allowing for either cavities causing recirculation or bluff bodies protruding into the flow is expected to reduce blood damage.^13,23^

The potential onset of coagulation/platelet activation may lead to the formation of thrombi at the outlet, and consequent risk of embolization to the patient. This risk is also present for micro-emboli created earlier in the circuit, before the ML, if the diameter is less than the distance between ML fibers (~200 μm), allowing emboli to pass through. This may lead to increased patient morbidity, for example, cerebral infarction or intracranial hemorrhage.^7^

### Limitations

The geometry was reconstructed from surface scanning and caliper measurements; therefore, some inaccuracies regarding the exact geometrical dimensions can be present. However, the results will not be affected as the recirculation zones and shear layers described in this work will still develop.

## Conclusions

Our study showed that the flow structures developing in this particular ML outlet were undesirable and would benefit from design revision. A review of devices used for ECMO is warranted, focusing on features such as auxiliary objects protruding into the bloodstream. Review is important to achieve increased understanding for improved general device design and, consequently, reduced risk of blood trauma and coagulation activation.

## Acknowledgments

The computations were enabled by resources provided by the National Academic Infrastructure for Supercomputing in Sweden (NAISS), partially funded by the Swedish Research Council through grant agreement no. 2022-06725. For sample preparation of the blood clot and the scanning electron microscopy measurements, the authors acknowledge the facilities and technical assistance of the Umeå Centre for Electron Microscopy (UCEM) at the Chemical Biological Centre (KBC), Umeå University, a part of the National Microscopy Infrastructure NMI (VR-RFI 2019-00217). The authors also acknowledge the ECMO Centre Karolinska, Astrid Lindgren’s Children’s Hospital, Karolinska University Hospital, Stockholm, for extracting the thrombus. Monica Norrby at Div. of Lightweight Structures, KTH Dept. Engineering Mechanics is acknowledged for generating the first version of the ML outlet CAD geometry.

## Supplementary Material


