# Supplementary material for: Protruding Objects in the Membrane Lung Outlet May Increase Thrombogenicity: Fluid Dynamical Insights
Source: ASAIO J. 2025 Aug 26;72(5):411–7. doi: 10.1097/MAT.0000000000002533 (PMC13108430; doi:10.1097/MAT.0000000000002533)
Supplement: Supplementary file 1 [file mat-72-411-s001.pdf]

SUPPLEMENTARY MATERIAL

Protruding Objects in the Membrane Lung Outlet May Increase Thrombogenicity: Fluid Dynamical Insights

Frida Nilsson[1], Monica Emendi[1], Lars Mikael Broman[2,3], Lisa Prahl Wittberg[1]

1. FLOW, Dept. Engineering Mechanics, KTH, Stockholm, Sweden
2. ECMO Centre Karolinska, Astrid Lindgren Children's Hospital, Karolinska University Hospital, Solna, Sweden
3. Dept. of Physiology and Pharmacology, Karolinska Institutet, Stockholm, Sweden

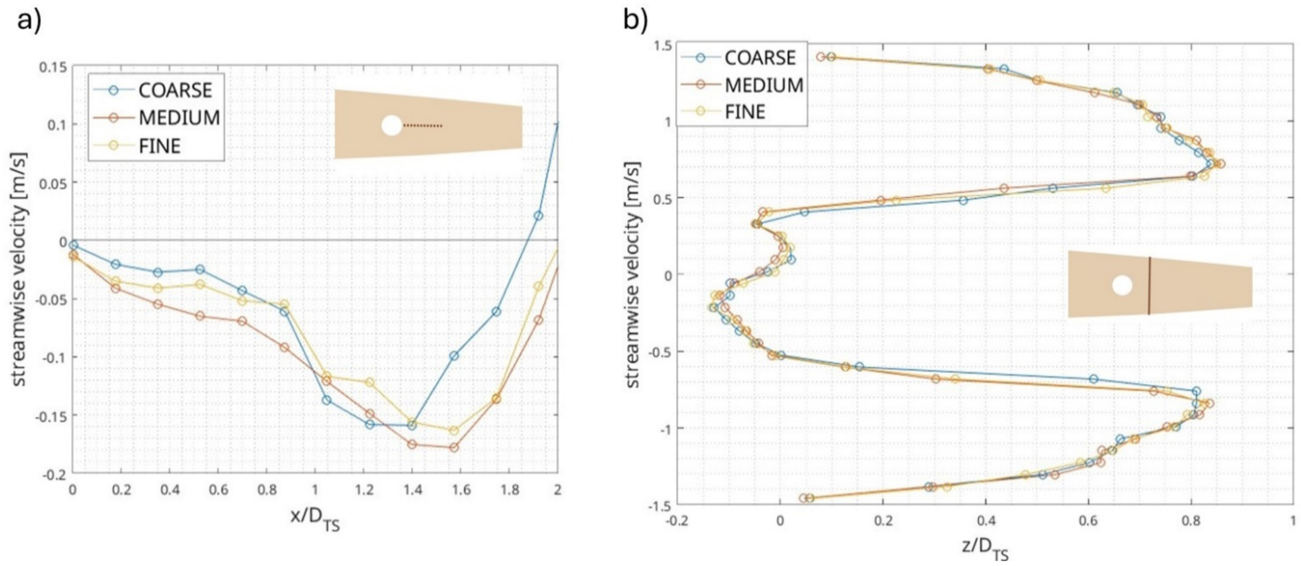

Figure S1: Grid convergence study of probe lines for the for the 4L/min case. Comparison of averaged streamwise velocity of a) a line probe along the wake of the temperature sensor and b) a line probe through the cross section of the wake.

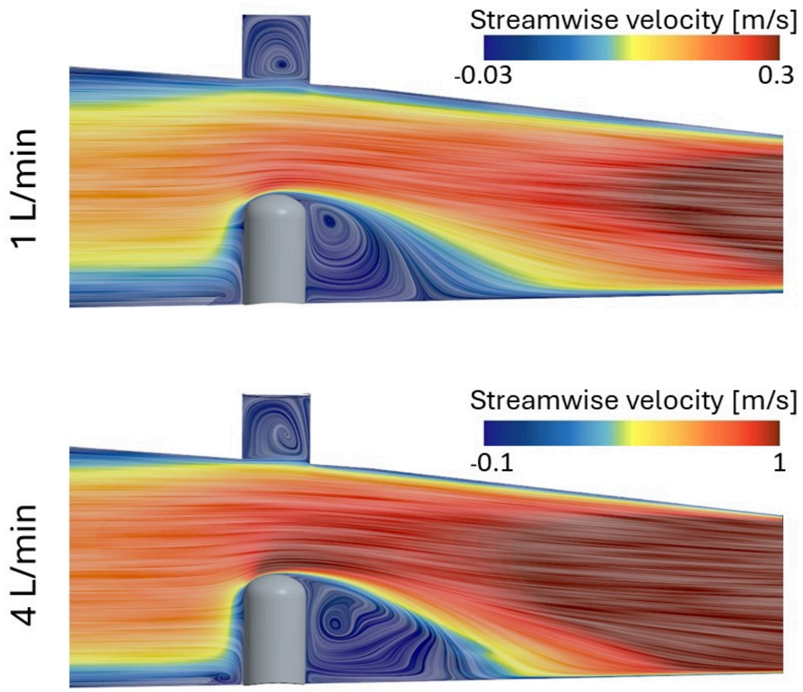

Figure S2: Averaged streamwise velocity, with line integral convolution of the velocity field. The recirculation areas behind the temperature sensor and in the Luer-lock port are clearly visible from this view.

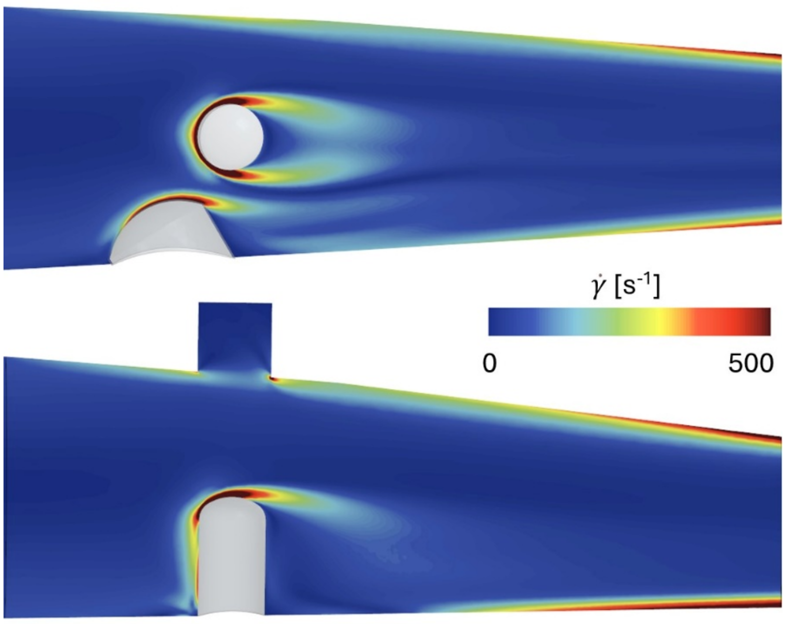

Figure S3: Average values of the fluid strain rate, $\dot{\gamma}$, on two perpendicular planes for the 1 L/min flow case.

Quemada viscosity model vs. Newtonian

A comparison of the difference in velocity obtained with the non-Newtonian Quemada model for blood viscosity and the Newtonian (constant viscosity) model used in this paper. For the 1L/min case, the largest differences were observed for the streamwise component in the wake behind the temperature sensor and inside the Luer-lock, with $\Delta u = u_{newtonian} - u_{quemada}$ and $\Delta u_{rel} = \frac{\Delta u}{u_{newtonian}} * 100$.

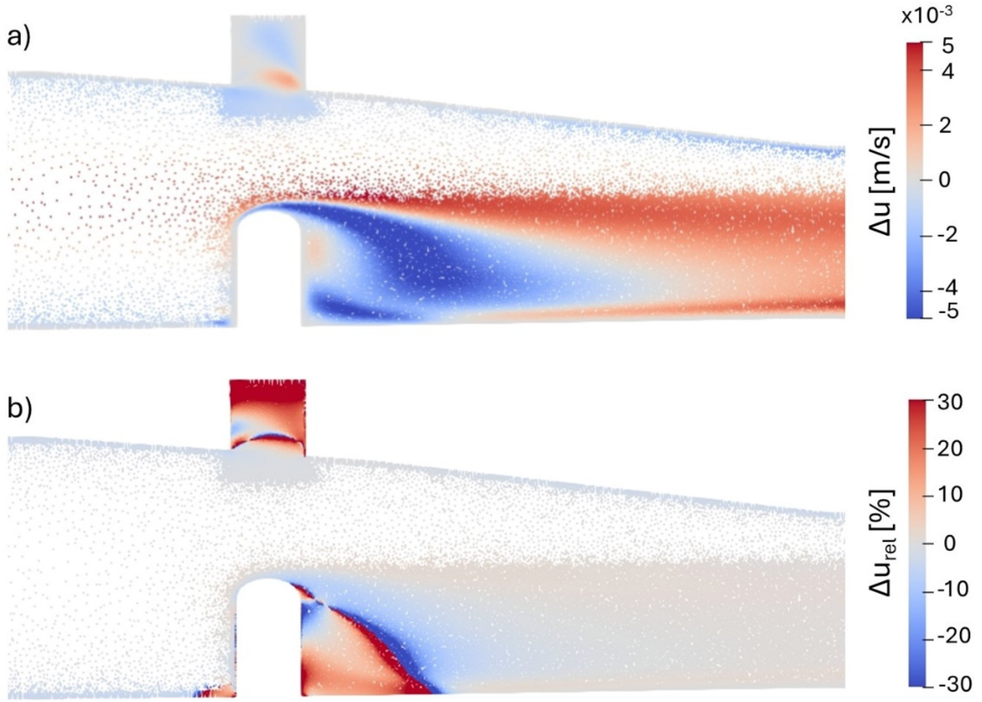

Figure S4: a) Difference in streamwise velocity, Δu, between the simulations with Quemada vs Newtonian viscosity model. b) Relative difference of streamwise velocity, Δu_{rel} between the simulations with Quemada vs Newtonian viscosity model. Figures refer to the 1 L/min case.
